# Comparison between three types of needles for endoscopic ultrasound-guided tissue acquisition of pancreatic solid masses: a multicenter observational study

**DOI:** 10.1038/s41598-023-30920-5

**Published:** 2023-03-04

**Authors:** Min Jae Yang, Jaihwan Kim, Se Woo Park, Jae Hee Cho, Eui Joo Kim, Yun Nah Lee, Dong Wook Lee, Chan Hyuk Park, Sang Soo Lee

**Affiliations:** 1 Department of Gastroenterology, Ajou University School of Medicine, Suwon, Korea; 2 Division of Gastroenterology, Department of Internal Medicine, Seoul National University Bundang Hospital, Seoul National University College of Medicine, Seongnam, Korea; 3 Division of Gastroenterology, Department of Internal Medicine, Hallym University Dongtan Sacred Heart Hospital, Hallym University College of Medicine, 7, Keunjaebong-Gil, Hwaseong-Si, 18450 Gyeonggi-Do Korea; 4 Division of Gastroenterology, Department of Internal Medicine, Gangnam Severance Hospital, Yonsei University College of Medicine, Seoul, Korea; 5 Division of Gastroenterology, Department of Internal Medicine, Gil Medical Center, Gachon University College of Medicine, Incheon, Korea; 6 Division of Gastroenterology, Department of Internal Medicine, SoonChunHyang University School of Medicine, Bucheon, Republic of Korea; 7 Department of Internal Medicine, School of Medicine, Kyungpook National University, Daegu, Korea; 8 Division of Gastroenterology, Department of Internal Medicine, Hanyang University Guri Hospital, Hanyang University College of Medicine, Guri, Korea; 9 Department of Gastroenterology, Asan Medical Center, University of Ulsan College of Medicine, Seoul, Korea

**Keywords:** Pancreas, Pancreatic cancer, Pancreatic cancer

## Abstract

It is debatable which needle has clear superiority of diagnostic performance in endoscopic ultrasound (EUS)-guided fine needle biopsy (FNB) of solid pancreatic masses. This study aimed to compare the performance of three needles and determine the variables that affect diagnostic accuracy. From March 2014 to May 2020, 746 patients with solid pancreatic masses who underwent EUS-FNB using three types of needles (Franseen needle, Menghini-tip needle, and Reverse-bevel needle) were retrospectively reviewed. Multivariate analysis using a logistic regression model was used to identify factors related to diagnostic accuracy. There were significant differences between the groups regarding the procurement rate of the histologic and optimal quality cores (Franseen vs. Menghini-tip vs. Reverse-bevel: 98.0% [192/196] vs. 85.8% [97/113] vs. 91.9% [331/360], *P* < 0.001 and 95.4% [187/196] vs. 65.5% [74/113] vs. 88.3% [318/360], *P* < 0.001, respectively). The sensitivity and accuracy using histologic samples were 95.03% and 95.92% for Franseen, 82.67% and 88.50% for Menghini-tip, and 82.61% and 85.56% for Reverse-bevel needles, respectively. In direct comparison between the needles using histologic samples, the Franseen needle showed significantly superior accuracy than the Menghini-tip (*P* = 0.018) and Reverse-bevel needles (*P* < 0.001). Multivariate analysis indicated that tumor size ≥ 2 cm (odds ratio [OR] 5.36, 95% confidence interval [CI] 3.40–8.47, *P* < 0.001) and fanning technique (OR 1.70, 95% CI 1.00–2.86, *P* = 0.047) were significantly associated with an accurate diagnosis. EUS-FNB using the Franseen needle enables the acquisition of a larger and more adequate histologic core tissue and achieves an accurate histological diagnosis when using the fanning technique.

## Introduction

Although endoscopic ultrasound (EUS)-guided tissue acquisition, including fine-needle aspiration (FNA) and fine-needle biopsy (FNB), is a standard modality for establishing a conclusive diagnosis and individualized therapeutic plan for pancreatic solid tumors^1^, the diagnostic performance has been reported to have a wide range according to the needle type. Several modified novel needles specially designed to obtain histologic cores with intact architecture have been announced recently to overcome these limitations of specific needle types^2^. These devices, collectively called FNB needles, have been equipped with the unique shape of a needle tip, which has either a side-fenestrated slot (core trap) or a special geometry of the cutting tip, while standard needles without these reinforcement geometries are classified as FNA needles^3^.

Initially, a new FNB needle with a reversed-bevel system as a side-fenestrated opening on the needle shaft was developed and is currently available as three gauges (19, 22, and 25) in the market. Theoretically, it can obtain the core tissue by hooking, cutting, and trapping it into the needle during the to-and-fro movement. Despite the hypothetical belief that the reversed-bevel design would yield a large piece of core tissue preserving the histological architecture, a special maneuver (e.g., scraping by momentary pulling force) during the to-and-fro movement might be required because it does not have a built-in cutting system^2^. More recently, the Franseen needle, which has a novel design of a crown tip with three-plane symmetric cutting edges, has been developed to facilitate the acquisition of larger core tissues.

Although recent clinical guidelines^3,4^ suggested that any specific type of needle, including FNA or FNB needle, does not guarantee superior diagnostic accuracy than others in EUS-guided tissue acquisition for pancreatic solid tumors, FNB needles tend to be customarily used to obtain adequate sampling tissue for differentiation of various tumors by immunohistochemistry (IHC) staining^5,6^.

Determining the optimal type of needle for accurate diagnosis, especially in the absence of rapid on-site cytological evaluation (ROSE), is critical for EUS-guided tissue acquisition for pancreatic solid tumors; however, there is little conclusive information regarding the relative diagnostic performance through comparison with various types of needles. Therefore, to provide more evidence on this topic, we compared the diagnostic performance according to the type of needle (Franseen needle vs. Reversed-bevel needle vs. standard FNA needle) and determined the variables that affect the diagnostic yield of malignancy of EUS-guided tissue acquisition for pancreatic solid tumors.

## Methods

### Patients

This was a multicenter retrospective study conducted at Ajou University Hospital, Hallym University Dongtan Sacred Heart Hospital, and Seoul National University Bundang Hospital. Consecutive patients who underwent either EUS-FNB or EUS-FNA for solid pancreatic tumors were included in this study. Patients with only a cystic component in the masses without a solid component suspected of malignant transformation were excluded. Demographic, clinical, and endoscopic data were extracted from a computerized clinical information system for the previous 7 years (from March 2014 to May 2020). Institutional Review Board approval was obtained from Hallym University Dongtan Sacred Heart Hospital (IRB file no: 2021-08-011). Furthermore, all procedures followed have been performed in accordance with the ethical standards laid down in the Declaration of Helsinki. The need for informed consent was waived due to the retrospective nature of this study by the Institutional Review Board of the Ethics Committee of Hallym University Dongtan Sacred Heart Hospital.

### Endoscopic procedures for EUS-FNA or FNB

**Figure 1:**
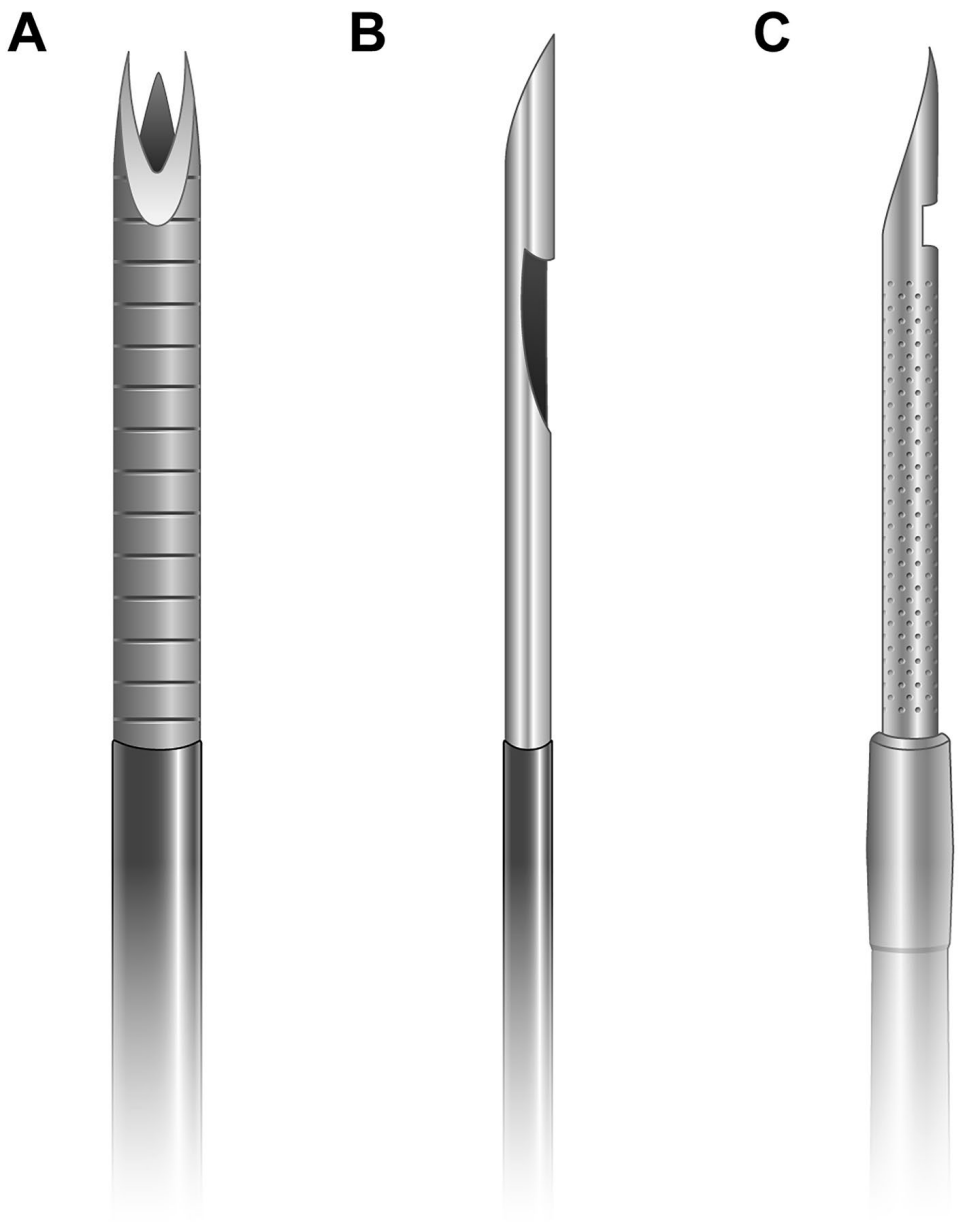
(**A**) The Franseen needle has a novel design of a crown tip with three-plane symmetric cutting edges. (**B**) The Reverse-bevel needle has a reversed-bevel system as a side-fenestrated opening on the needle shaft. (**C**) The Menghini-tip needle has a tapered bevel edge that facilitates the tissue being withdrawn into the lumen. Although it has a side port, it is classified as FNA needles because the side port of this needle system does not have any reinforcement geometries for cutting the tissue.

All procedures were performed with a linear array echoendoscope (EG-530UT2, Fujifilm Medical Systems, Tokyo, Japan or UCT 260, Olympus Medical Systems, Tokyo, Japan) by experienced endosonographers (M.J.Y, J.K, and S.W.P) with more than 100 cases of EUS-FNA or FNB per year under a well-established standard protocol^7^. EUS-FNB was attempted with a biopsy needle (Franseen needle; Acquire; Boston Scientific (Fig. 1A) or Reverse-bevel needle; EchoTip ProCore; Cook Endoscopy (Fig. 1B)) or EUS-FNA with a Menghini-tip needle (EZ shot3, Olympus Medical Systems, Tokyo, Japan (Fig. 1C)) as directed by the characteristics and location of pancreatic tumors and endosonographer’s preference. Furthermore, the size of the needle used (22-gauge or 25-gauge) was chosen at the discretion of the endosonographers. After confirming the absence of intervening vasculature on the expected needle track using the color Doppler, a needle mounted with a stylet was used to create a puncture at either the stomach for body/tail lesions or the duodenum for head/uncinate process lesions. After puncturing the lesion, the stylet was withdrawn, and approximately 10–20 back-and-forth movements were performed within the lesion during each needle passage with continuous suction using a 10–20 mL syringe provided by the manufacturers. Detailly, 10 mL negative suction was applied to the Reverse-bevel needle, while 20 mL negative suction was applied to the EZ shot3 and Franseen needles. In addition, EUS-FNA or FNB was repeated until sufficient visible core tissue was obtained, although the optimal number of needle passes was decided at the discretion of the endosonographers^8^. ROSE was not available in any institution.

### Cytopathological analysis using each FNB needle

In this study, cytological and histological evaluations were performed by a single cytopathologist experienced in pancreatology at each institution. The samples obtained from each passage by advancing the stylet within the needle assembly were fixed entirely into 50% ethanol for cell block evaluation and formalin bottles for histologic evaluation^9^. The prepared histological samples fixed in formalin solutions were re-processed in cassette form, embedded in paraffin, and prepared in hematoxylin and eosin (H & E) stain for evaluation by the same pathologist. When necessary, special staining such as IHC staining was applied to differentiate between tumor cells and regenerative atypia or atypical tumors such as lymphoma, metastatic carcinoma, solid pseudopapillary tumors, or even neuroendocrine tumors. If a histological core was not obtained, the cytopathologist processed the same material as the cell block for cytological analysis.

### Definition of outcomes and reference criteria for the diagnosis of benign or malignant pancreatic masses

The primary outcome of this study was the diagnostic performance of the needles. The secondary outcomes were defined as the procurement rates of histologic cores considered to be of optimal quality for histological evaluation according to the needles used for the EUS-FNB procedure, procedure-related adverse events, and variables that affected the diagnostic accuracy in a logistic regression model. In detail, sufficient visible core tissue was defined as whitish or reddish pieces of tissue with apparent bulk on the filter paper or slide glass^10^.

A final diagnosis was established as either a malignant or benign mass based on one of the following reference criteria: (a) a definite diagnosis based on the evaluation of surgically resected permanent specimens from operated patients, (b) disease-specific mortality, and (c) no evidence of disease regression or progression during the longer than 6-month follow-up periods according to clinical and radiological workups only in cases of suspected benign disease at the time of the EUS-FNB^7,11,12^. In the initial categorization as definite or suspected malignancy based on cell block or histologic analysis by EUS-FNA or FNB, cases confirmed as malignancy in the final diagnosis were considered as true positives, while lesions that were finally diagnosed as benign diseases after the clinical follow-up were considered false positives. Similarly, if the initial benign results were finally diagnosed as benign diseases, they were considered as true negatives, while those confirmed as malignancies in the final diagnosis were considered as false negatives. Furthermore, non-diagnostic results, including insufficient samples, were considered false negatives because the procedure failed to provide a diagnosis^13^.

### Statistical analysis

Continuous variables were presented as mean and standard deviation and were compared using Student’s t-test. Categorical variables were presented as numbers (percentages) and were compared using the χ^2^ test. Univariate and multivariate logistic regression models were applied to assess the relationship between clinical and endoscopic variables, including needle type, with an accurate diagnosis. The variables found to be significant (*P* < 0.20) in the univariate model were entered into the multivariate logistic regression model. The diagnostic sensitivity, specificity, accuracy, positive predictive value (PPV), and negative predictive value (NPV) were evaluated for all needle types. All reported *P*-values were two-sided, and *P*-values < 0.05 were considered statistically significant. All statistical analyses were conducted using the statistical software R (version 3.3.3; R Foundation for Statistical Computing, Vienna, Austria).

## Results

### Study population and baseline characteristics

**Figure 2:**
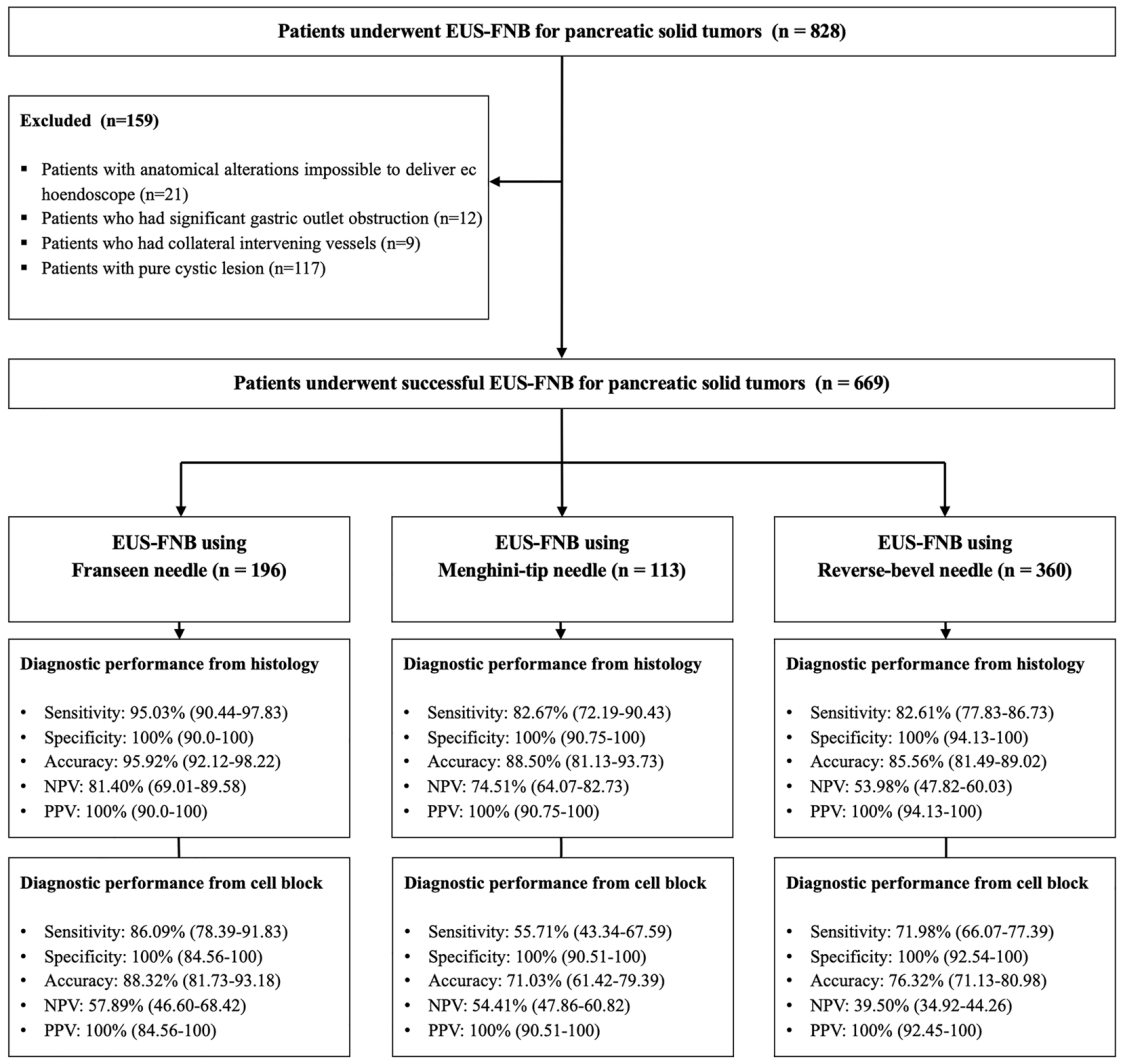
Flow diagram of patients throughout the study.

During the study period, 828 patients underwent EUS-FNB for solid pancreatic tumors (Fig. 2). Patients with anatomical alterations, which made it impossible to deliver an echoendoscope (n = 21), those who had significant gastric outlet obstruction (n = 12), those who had collateral intervening vessels, which made it impossible to puncture with a needle (n = 9), and those with pure cystic lesions without a solid component that was only possible for aspiration and not tissue acquisition (n = 117) were excluded. Overall, 669 patients were included in the analyses and divided into three groups: Franseen (n = 196), Menghini-tip (n = 113), and Reverse-bevel (n = 360).

Table 1 presents the baseline characteristics of the patients according to each group. The mean age of the Franseen group was significantly higher than that of other groups (Franseen vs. Menghini-tip vs. Reverse-bevel group: 66.3 ± 12.0 vs. 62.5 ± 15.2 vs. 65.0 ± 12.0, respectively; *P* = 0.039). The proportion of male patients and body mass index did not differ between the groups. Additionally, a 22-gauge needle was predominantly used in the Franseen and Menghini-tip groups, while the 25-gauge needle was used frequently in the Reverse-bevel group with a significant difference (*P* < 0.001). In the three groups, a suspected malignant mass was the most common indication for EUS-FNA/B, with a significant difference (*P* = 0.008). The mass was most frequently located in the pancreatic head (39.3%, 45.1%, and 51.9% in the Franseen, Menghini-tip, and Reverse-bevel groups, respectively) with significant intergroup differences. The mean size of the lesion was 30.3 mm in the Franseen group, 36.3 mm in the Menghini-tip group, and 29.9 mm in the Reverse-bevel group, with significant intergroup differences.

**Table 1:** Characteristics of patients.

	Franseen n = 196	Menghini-tip n = 113	Reverse-bevel n = 360	*P* value
Age, year, mean ± SD	66.3 ± 12.0	62.5 ± 15.2	65.0 ± 12.0	0.039
Sex (Male), n (%)	111 (56.6%)	64 (56.6%)	192 (53.3%)	0.694
BMI, kg/m^2^, mean ± SD	23.1 ± 3.5	22.5 ± 4.3	22.6 ± 3.3	0.202
Needle diameter, n (%)				< 0.001
22-gauge	180 (91.8%)	113 (100.0%)	152 (42.2%)	
25-gauge	16 (8.2%)	0 (0.0%)	208 (57.8%)	
Indication of TA, n (%)				0.008
Suspected benign mass	20 (10.2%)	25 (22.1%)	44 (12.2%)	
Suspected malignant mass	176 (89.8%)	88 (77.9%)	316 (87.8%)	
Location of lesion, n (%)				0.048
Uncinate process	20 (10.2%)	9 (8.0%)	41 (11.4%)	
Head	77 (39.3%)	51 (45.1%)	187 (51.9%)	
Body	72 (36.7%)	40 (35.4%)	91 (25.3%)	
Tail	27 (13.8%)	13 (11.5%)	41 (11.4%)	
Maximal size of lesion, mm, mean ± SD	30.3 ± 10.9	36.3 ± 22.3	29.9 ± 10.5	< 0.001
Initial laboratory findings, median [IQR]				
WBC, mm^3^	9750.0 [6290.0–12,600.0]	6400.0 [4700.0–7900.0]	8700.0 [5735.0–12,500.0]	< 0.001
Hemoglobin, g/dL	12.6 [11.4–13.7]	12.6 [11.1–14.1]	12.6 [11.5–13.6]	0.831
Platelet, × 1000/mm^3^	221.0 [174.0–268.0]	230.0 [178.0–266.0]	222.0 [179.0–275.5]	0.921
AST, IU/L	32.5 [19.0–116.0]	24.0 [19.0–42.0]	33.0 [21.0–122.0]	0.012
ALT, IU/L	30.0 [15.0–126.0]	25.0 [15.0–48.0]	34.0 [16.5–149.5]	0.053
ALP, IU/L	101.0 [65.0–306.0]	86.0 [62.0–165.0]	117.5 [71.0–364.5]	0.001
GGT, IU/L	96.0 [21.0–592.0]	48.0 [17.0–198.0]	108.0 [24.0–634.0]	0.002
Total bilirubin, mg/dL	0.7 [0.4–6.0]	0.6 [0.4–1.0]	0.8 [0.4–7.1]	0.003
Amylase, IU/L	63.0 [38.0–93.0]	62.0 [47.0–104.0]	66.5 [44.0–114.0]	0.608

SD, standard deviation; BMI, body mass index; WBC, white blood cell; AST, aspartate aminotransferase; IU, international unit; ALT, alanine transaminase; ALP, alkaline phosphatase; GGT, gamma-glutamyltransferase.

### Procedure-related findings between the three groups

Procedure-related outcomes are presented in Table 2. The transduodenal approach was greater than the transgastric approach in all three groups because the most common location of the lesions was the head in all groups. Moreover, the number of needle passes was higher in the Reverse-bevel group than in the two other groups (Franseen vs. Menghini-tip vs. Reverse-bevel group: 2.8 ± 1.0 vs. 2.9 ± 0.5 vs. 3.4 ± 1.3, *P* < 0.001). Continuous suction with negative pressure using a syringe was applied during EUS-FNA/B for all patients in the three groups. The Fanning technique was applied to all patients in the Franseen group, while it was performed for only 53.1% of patients in the Menghini-tip group and 82.2% of patients in the Reverse-bevel group (*P* < 0.001) with significant intergroup differences.

**Table 2:** Comparison of procedure-related outcomes between the three groups.

	Franseen n = 196	Menghini-tip n = 113	Reverse-bevel n = 360	*P* value
Approaches, n (%)				0.004
Trans-duodenal	96 (49.0%)	60 (53.1%)	227 (63.1%)	
Trans-gastric	100 (51.0%)	53 (46.9%)	133 (36.9%)	
Number of passes, mean ± SD	2.8 ± 1.0	2.9 ± 0.5	3.4 ± 1.3	< 0.001
Application of suction, n (%)	196 (100.0%)	113 (100.0%)	360 (100.0%)	
Amounts of suction, n (%)				< 0.001
10 ml	0 (0.0%)	0 (0.0%)	235 (65.3%)	
20 ml	196 (100.0%)	113 (100.0%)	125 (34.7%)	
Application of stylet, n (%)	145 (74.0%)	113 (100.0%)	275 (76.4%)	< 0.001
Fanning technique, n (%)	196 (100.0%)	60 (53.1%)	296 (82.2%)	< 0.001
Technical success, n (%)	196 (100.0%)	113 (100.0%)	360 (100.0%)	
Adverse events, n (%)				
Immediate bleeding	1 (0.5%)	2 (1.8%)	5 (1.4%)	0.546
Delayed bleeding	0 (0.0%)	0 (0.0%)	0 (0.0%)	
Pancreatitis	3 (1.5%)	0 (0.0%)	2 (0.6%)	0.266
Infection	0 (0.0%)	0 (0.0%)	0 (0.0%)	
Any cardiopulmonary distress. during procedure	1 (0.5%)	1 (0.9%)	0 (0.0%)	0.263
Final diagnosis, n (%)				0.010
Inflammatory tumor	17 (8.7%)	19 (16.8%)	29 (8.1%)	
Benign tumor	18 (9.2%)	19 (16.8%)	32 (8.9%)	
PNEC	1 (0.5%)	2 (1.8%)	1 (0.3%)	
Lymphoma	0 (0.0%)	1 (0.9%)	3 (0.8%)	
Metastatic carcinoma	0 (0.0%)	1 (0.9%)	3 (0.8%)	
Ductal adenocarcinoma	160 (81.6%)	71 (62.8%)	292 (81.1%)	
Presence of histologic core, n (%)	192 (98.0%)	97 (85.8%)	331 (91.9%)	< 0.001
Possibility for IHC stain, n (%)	187 (95.4%)	74 (65.5%)	318 (88.3%)	< 0.001

IHC, immunohistochemical; NET_,_ neuroendocrine tumor; IPMN, intraductal papillary mucinous neoplasm.

No technical difficulties were encountered in either group, even when transduodenal passes were performed. Regarding procedure-related adverse events, only eight patients (8/669, 1.2%) in the entire cohort had immediate bleeding as an adverse event, and all patients in each group were managed by clipping several times on the same endoscopic session. In addition, only five patients developed mild acute pancreatitis that required hospitalization within 3 days (1.5% in the Franseen group and 0.6% in the Reverse-bevel group, *P* = 0.266). No patient in each group experienced significantly delayed bleeding or infectious adverse events. Histological core tissue was significantly higher in the Franseen than Menghini-tip and Revers-bevel groups (Franseen vs. Menghini-tip vs. Reverse-bevel group: 98.0% vs. 85.8% vs. 91.9%, *P* < 0.001). Furthermore, core biopsy specimens that were adequate for IHC analysis were obtained in 95.4% of cases using the Franseen needle, 65.5% for the Menghini-tip needle, and 88.3% for the Reverse-bevel needle (*P* < 0.001). The most common final diagnosis in the three groups was ductal adenocarcinoma.

### Diagnostic performance of each needle for EUS-FNA/B

Diagnostic performance was calculated in two different ways using the methods of specimen processing. On the initial diagnosis by cell block evaluation, the Franseen needle had 86.09% sensitivity, 100.0% specificity, a PPV of 100%, and an NPV of 57.89%, while the Menghini-tip needle had 55.71% sensitivity, 100.0% specificity, a PPV of 100.0%, and an NPV of 54.41% (Table 3). In addition, the Reverse-bevel needle had 71.98% sensitivity, 100.0% specificity, a PPV of 100%, and an NPV of 39.50%. Moreover, the diagnostic accuracies for the Franseen, Menghini-tip, and Reverse-bevel needles were 88.32%, 71.03%, and 76.32%, respectively. On the initial diagnosis by histologic evaluation, the Franseen needle provided improved diagnostic performance (95.03% sensitivity, 100.0% specificity, a PPV of 100.0%, and an NPV of 81.40%) with an accuracy of 95.92%; the Menghini-tip needle provided diagnostic performance (82.67% sensitivity, 100.0% specificity, a PPV of 100.0%. and an NPV of 74.51%) with an accuracy of 88.50%; while the Reverse-bevel needle showed a diagnostic performance (82.61% sensitivity, 100.0% specificity, a PPV of 100.0%, and an NPV of 53.98%) with an accuracy of 85.56%.

**Table 3:** Diagnostic performance according to the needles.

	Franseen, % (95% Cl)		Menghini-tip, % (95% Cl)		Reverse-bevel, % (95% Cl)	
	Cell block	Histology	Cell block	Histology	Cell block	Histology
Sensitivity	86.09% (78.39–91.83)	95.03% (90.44–97.83)	55.71% (43.34–67.59)	82.67% (72.19–90.43)	71.98% (66.07–77.39)	82.61% (77.83–86.73)
Specificity	100.00% (84.56–100.00)	100.00% (90.00–100.00)	100.00% (90.51–100.00)	100.00% (90.75–100.00)	100.00% (92.45–100.00)	100.00% (94.13–100.00)
Accuracy	88.32% (81.73–93.18)	95.92% (92.12–98.22)	71.03% (61.42–79.39)	88.50% (81.13–93.73)	76.32% (71.13–80.98)	85.56% (81.49–89.02)
Negative predictive value	57.89% (46.60–68.42)	81.40% (69.01–89.58)	54.41% (47.86–60.82)	74.51% (64.07–82.73)	39.50% (34.92–44.26)	53.98% (47.82–60.03)
Positive predictive value	100.00% (84.56–100.00)	100.00% (90.00–100.00)	100.00% (90.51–100.00)	100.00% (90.75–100.00)	100.00% (92.45–100.00)	100.00% (94.13–100.00)

Cl, confidence interval.

**Figure 3:**
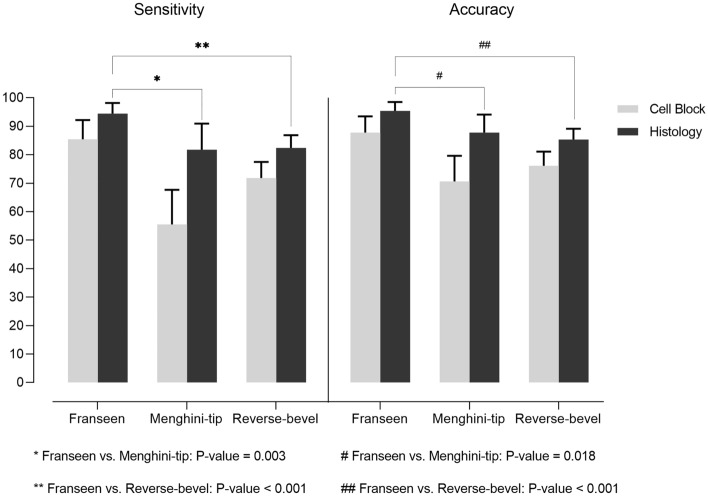
Comparison of diagnostic performance according to the needles.

In direct comparison of diagnostic performances using histologic samples between the needles, the Franseen needle showed significantly superior sensitivity than the Menghini-tip (*P* = 0.003) and Reverse-bevel needles (*P* < 0.001) (Fig. 3). Furthermore, the Franseen needle also showed significantly superior accuracy than the Menghini-tip (*P* = 0.018) and Reverse-bevel needles (*P* < 0.001).

### Variables associated with diagnostic accuracy

Univariate and multivariate analyses were performed using logistic regression models to identify the factors associated with diagnostic accuracy (Table 4). Tumor size > 2 cm (odds ratio [OR]: 5.36, 95% confidence interval [CI]: 3.40–8.47) and the application of the fanning technique (OR: 1.70, 95% CI: 1.00–2.86) were significantly associated with an accurate diagnosis in the multivariable analysis. Furthermore, the Menghini-tip needle was identified as an associate variable for poor accurate diagnosis (OR: 0.54, 95% CI: 0.29–1.00, *P* = 0.050) with marginal significance. Furthermore, FNB needles, including Franseen and Reverse-bevel needles, were significantly associated with accurate diagnosis compared to FNA needles (Menghini-tip needle) (OR: 2.03, 95% CI: 1.21–3.38, *P* = 0.007) (Table S1).

**Table 4:** Variables for accurate diagnosis according to logistic regression models.

Variable	Univariate analysis		Multivariate analysis	
	OR (95% CI)	*P*-value	OR (95% CI)	*P*-value
Menghini-tip (vs. Franseen)	0.43 (0.25–0.73)	0.002	0.54 (0.29–1.00)	0.050
Reverse-bevel (vs. Franseen)	1.07 (0.67–1.68)	0.786	1.18 (0.71–1.94)	0.509
25-gauge (vs. 22-gauge)	1.23 (0.82–1.87)	0.320		
Tumor size ≥ 2 cm (vs. < 2 cm)	5.36 (3.43–8.40)	< 0.001	5.36 (3.40–8.47)	< 0.001
Trans-gastric (vs. Trans-duodenal)	0.97 (0.66–1.43)	0.889		
Needle pass ≥ 4 (vs. < 4)	1.83 (1.14–3.04)	0.152		
Fanning technique (vs. no fanning)	2.17 (1.38–3.38)	< 0.001	1.70 (1.00–2.86)	0.047
20 ml of suction (vs. 10 ml of suction)	1.08 (0.73–1.60)	0.696		
Application of stylet (vs. no stylet)	0.77 (0.46–1.25)	0.310		

OR, odds ratio; CI, confidence interval.

## Discussion

According to the development and improvement of new types of needles for EUS-FNA/B, many trials^2,14–19^ revealed that modified needles with reinforcement geometries on the tip provided superior diagnostic accuracy and histological core tissue procurement for pancreatic solid tumors. Our study is the first report that compared the three types of needles (Franseen needle, Reverse-bevel needle, and Menghini-tip needle) for adequacy and accuracy of samples from each needle. In our retrospective comparative study, EUS-FNB using a Franseen needle was associated with a higher procurement rate of histologic core tissue than Reverse-bevel needles and even Menghini-tip needles. Furthermore, the Franseen needle showed overwhelmingly improved diagnostic performance, including 95% sensitivity, 96% accuracy, and 81.4% NPV compared to other needle types. In the first preliminary report for Franseen needle, Bang et al. demonstrated that a unique crown tip with three-plane symmetrical cutting edges enables better targeting of lesions with lower penetration force and even greater tissue acquisition^20^. Thereafter, they prospectively compared the cellularity and diagnostic accuracy of four types of needles, including the Franseen, Reverse-bevel, and Menghini-tip needles^21^. In this study, the authors concluded that the Franseen needle showed the highest degree of cellularity for pancreatic solid tumors, although the number of patients assigned to each group was as relatively small as 32–33 patients. Furthermore, the Franseen needle showed the highest diagnostic accuracy of 92.7%, while those of the Menghini-tip and Reverse-bevel needles were 74.7% and 67.7%, respectively. These results were entirely consistent with our results and our hypothesis that the pathological outcomes of EUS-FNA/FNB are totally dependent on the type of needle used for tissue sampling.

Notably, the fanning technique was an independent favoring factor for accurate diagnosis, although an initial study by Bang et al. failed to verify the significant impact of the technique on diagnostic performance^22^. Recent clinical guideline strongly suggests that the fanning technique for EUS-guided tissue acquisition offers technically acceptable feasibility and superior diagnostic outcomes, including fewer needle passes required to establish a definite diagnosis than the standard technique^3^. Thus, our study reinforces the need for the fanning technique for routine application for EUS-FNA/FNB from clinical guidelines: the fanning technique increases diagnostic accuracy. Theoretically, the application of the fanning technique can increase the likelihood of achieving an accurate diagnosis, thereby reducing the possibility of inconclusive results without additional risk of adverse events or medical costs.

Our study has several valuable implications. First, EUS-FNA/FNB using a Franseen needle might reduce the need for ROSE because its sensitivity and diagnostic accuracy for malignancy by histologic evaluation exceeded 95% without ROSE in all cases. Although ROSE provides a high per-case adequacy of a sample with fewer number of needle passes^23^, recent observational data have reported conflicting results, in which ROSE may not be associated with an improvement in diagnostic yield, including accuracy and sensitivity^24^. Second, optimal histologic core tissue, which can be achieved for special staining such as IHC, was obtained in 95% of the Franseen needle. Acquiring reliable and sufficient tissue can provide preserved histologic architecture for special staining and even molecular profiling, which is essential for the differential diagnosis of other pancreatic tumors or inflammatory masses and personalized anti-cancer therapy^25^. Third, although recent guidelines^3^ recommended that a minimum of four passes is required to achieve an accurate diagnosis and more than four passes may be required for tumors less than 2 cm, in our study, superior diagnostic performance was achieved with fewer passes using a Franseen needle than Reverse-bevel or Menghini-tip needles. Therefore, in institutions relying on the Reverse-bevel and Menghini-tip needles, we carefully recommend conducting three or four passes to achieve an accurate diagnosis.

Although our study is the first to evaluate the diagnostic performance of the three types of needles for EUS-FNA/B in a large cohort, it had some limitations that might have influenced our final conclusions. First, several technical biases, including the application of suction, stylet, or fanning methods, could not be completely avoided because of the retrospective nature of the study. In addition, retrospective data analysis may lack some information about important variables (information bias). Although we adjusted for potentially confounding variables, including needle type, needle diameter, tumor size, approaching route, number of needle passes, and hidden or unmeasured factors may have remained. Therefore, this study still has important limitations. We hope that this limitation can be overcome through randomized controlled trials. Second, the cytopathologic results were determined by a single pathologist at each center, which may have introduced observer bias with inter- and intra-observer variations. Third, our methodology for defining benign diseases has not been validated. Nevertheless, to avoid unnecessary surgery, our strategy, including clinical follow-up of ≥ 6 months with repeated workups, was relatively reasonable for defining benign disease, although admittedly, not ideal. Fourth, there might have been heterogeneity between the present study and many other studies regarding the definition of malignancy in which “highly suggestive” samples could be generally considered diagnostic and acceptable^26^. However, there might have been a discrepancy in diagnostic performance if highly suggestive samples were categorized as diagnostic of malignancy or not.

In conclusion, to establish the optimal needle and technique that can yield an accurate diagnosis, we believe that our suggestions of using the Franseen needle under the fanning technique may enable standardization of the practice of EUS-guided tissue acquisition in pancreatic cancer. Furthermore, FNB needles with any reinforcement geometries, instead of standard FNA needles, can be recommended regardless of the model and manufacturer of needles.

## Supplementary Information

Supplementary Information.

## Data Availability

The datasets generated during and/or analyzed during the current study are available from the corresponding author on reasonable request.

## References

[1] ASGE Standards of Practice Committee (2016). The role of endoscopy in the evaluation and management of patients with solid pancreatic neoplasia. Gastrointest. Endosc..

[2] Bang JY, Hawes R, Varadarajulu S (2016). A meta-analysis comparing ProCore and standard fine-needle aspiration needles for endoscopic ultrasound-guided tissue acquisition. Endoscopy.

[3] Chung MJ (2021). Clinical and technical guideline for endoscopic ultrasound (EUS)-guided tissue acquisition of pancreatic solid tumor: Korean society of gastrointestinal endoscopy (KSGE). Gut Liver.

[4] Polkowski M (2017). Technical aspects of endoscopic ultrasound (EUS)-guided sampling in gastroenterology: European Society of Gastrointestinal Endoscopy (ESGE) Technical Guideline—March 2017. Endoscopy.

[5] Dim DC (2014). The usefulness of S100P, mesothelin, fascin, prostate stem cell antigen, and 14-3-3 sigma in diagnosing pancreatic adenocarcinoma in cytological specimens obtained by endoscopic ultrasound guided fine-needle aspiration. Diagn. Cytopathol..

[6] Hasegawa T (2014). Evaluation of Ki-67 index in EUS-FNA specimens for the assessment of malignancy risk in pancreatic neuroendocrine tumors. Endoscopy.

[7] Park SW (2020). The diagnostic performance of novel torque technique for endoscopic ultrasound-guided tissue acquisition in solid pancreatic lesions: A prospective randomized controlled trial. J. Gastroenterol. Hepatol..

[8] Sun B, Yang X, Ping B, He Y, Zhang Z (2015). Impact of inconclusive endoscopic ultrasound-guided fine-needle aspiration results in the management and outcome of patients with solid pancreatic masses. Dig. Endosc..

[9] Matsubayashi H (2018). Pathological and molecular aspects to improve endoscopic ultrasonography-guided fine-needle aspiration from solid pancreatic lesions. Pancreas.

[10] Oh D (2019). The impact of macroscopic on-site evaluation using filter paper in EUS-guided fine-needle biopsy. Endosc. Ultrasound.

[11] Iglesias-Garcia J (2011). Feasibility and yield of a new EUS histology needle: Results from a multicenter, pooled, cohort study. Gastrointest. Endosc..

[12] Fabbri C (2011). Endoscopic ultrasound-guided fine needle aspiration with 22- and 25-gauge needles in solid pancreatic masses: A prospective comparative study with randomisation of needle sequence. Dig. Liver Dis..

[13] Park SW (2016). Prospective study for comparison of endoscopic ultrasound-guided tissue acquisition using 25- and 22-gauge core biopsy needles in solid pancreatic masses. PLoS ONE.

[14] Chong CCN (2018). EUS-FNA using 22G nitinol or ProCore needles without on-site cytopathology. Endosc. Ultrasound.

[15] Fujie S (2019). Comparison of the diagnostic yield of the standard 22-gauge needle and the new 20-gauge forward-bevel core biopsy needle for endoscopic ultrasound-guided tissue acquisition from pancreatic lesions. Gut Liver.

[16] Kandel P (2016). EUS-guided fine needle biopsy sampling using a novel fork-tip needle: A case-control study. Gastrointest. Endosc..

[17] Naveed M (2018). A Multicenter comparative trial of a novel EUS-guided core biopsy needle (SharkCore™) with the 22-gauge needle in patients with solid pancreatic mass lesions. Endosc. Ultrasound.

[18] Bang JY (2018). Randomized trial comparing the Franseen and Fork-tip needles for EUS-guided fine-needle biopsy sampling of solid pancreatic mass lesions. Gastrointest. Endosc..

[19] Mukai S (2019). A retrospective histological comparison of EUS-guided fine-needle biopsy using a novel franseen needle and a conventional end-cut type needle. Endosc. Ultrasound.

[20] Bang JY (2017). Endoscopic ultrasonography-guided biopsy using a Franseen needle design: Initial assessment. Dig. Endosc..

[21] Bang JY, Krall K, Jhala N (2021). Comparing needles and methods of endoscopic ultrasound-guided fine-needle biopsy to optimize specimen quality and diagnostic accuracy for patients with pancreatic masses in a randomized trial. Clin. Gastroenterol. Hepatol..

[22] Bang JY, Magee SH, Ramesh J, Trevino JM, Varadarajulu S (2013). Randomized trial comparing fanning with standard technique for endoscopic ultrasound-guided fine-needle aspiration of solid pancreatic mass lesions. Endoscopy.

[23] Matynia AP (2014). Impact of rapid on-site evaluation on the adequacy of endoscopic-ultrasound guided fine-needle aspiration of solid pancreatic lesions: A systematic review and meta-analysis. J. Gastroenterol. Hepatol..

[24] Kong F (2016). Rapid on-site evaluation does not improve endoscopic ultrasound-guided fine needle aspiration adequacy in pancreatic masses: A meta-analysis and systematic review. PLoS ONE.

[25] Biankin AV, Hudson TJ (2011). Somatic variation and cancer: Therapies lost in the mix. Hum. Genet..

[26] Nayar MK (2017). Comparison of the diagnostic performance of 2 core biopsy needles for EUS-guided tissue acquisition from solid pancreatic lesions. Gastrointest. Endosc..

