# Supplementary material for: Comparison between three types of needles for endoscopic ultrasound-guided tissue acquisition of pancreatic solid masses: a multicenter observational study
Source: Sci Rep. 2023 Mar 4;13:3677. doi: 10.1038/s41598-023-30920-5 (PMC9985625; doi:10.1038/s41598-023-30920-5)
Supplement: Supplementary file 1 — Supplementary Information. [file 41598_2023_30920_MOESM1_ESM.docx]

**Table S1. Variables for accurate diagnosis according to logistic regression models**

| Variable | Univariate analysis | |  | Multivariate analysis | |
| --- | --- | --- | --- | --- | --- |
|  | OR (95% CI) | P-value |  | OR (95% CI) | P-value |
| **FNB needle (vs. FNA needle)** | 2.43 (1.54-3.78) | 0.002 |  | 2.03 (1.21-3.38) | 0.007 |
| **25-gauge (vs. 22-gauge)** | 1.23 (0.82-1.87) | 0.320 |  |  |  |
| **Tumor size ≥2cm (vs. <2cm)** | 5.36 (3.43-8.40) | <0.001 |  | 5.27 (3.33-8.34) | <0.001 |
| **Trans-gastric (vs. Trans-duodenal)** | 0.97 (0.66-1.43) | 0.889 |  |  |  |
| **Needle pass ≥4 (vs. <4)** | 1.83 (1.14-3.04) | 0.152 |  | 1.23 (0.73-2.12) | 0.444 |
| **Fanning technique (vs. no fanning)** | 2.17 (1.38-3.38) | <0.001 |  | 1.58 (0.94-2.62) | 0.077 |
| **20ml of suction (vs. 10ml of suction)** | 1.08 (0.73-1.60) | 0.696 |  |  |  |
| **Application of stylet (vs. no stylet)** | 0.77 (0.46-1.25) | 0.310 |  |  |  |

OR, odds ratio; CI, confidence interval
